# A gene × gene interaction between DRD2 and DRD4 is associated with conduct disorder and antisocial behavior in males

**DOI:** 10.1186/1744-9081-3-30

**Published:** 2007-06-22

**Authors:** Kevin M Beaver, John Paul Wright, Matt DeLisi, Anthony Walsh, Michael G Vaughn, Danielle Boisvert, Jamie Vaske

**Affiliations:** 1 College of Criminology and Criminal Justice, Florida State University, Tallahassee, FL 32306-1127, USA; 2 Division of Criminal Justice, University of Cincinnati, Cincinnati, OH 45221-0389, USA; 3 Department of Sociology, Iowa State University, Ames, IA 50011-1070, USA; 4 Department of Criminal Justice Administration, Boise State University, Boise, ID 83725, USA; 5 School of Social Work, University of Pittsburgh, Pittsburgh, PA 15260, USA

## Abstract

**Background:**

Antisocial behaviors are complex polygenic phenotypes that are due to a multifactorial arrangement of genetic polymorphisms. Little empirical research, however, has been undertaken that examines gene × gene interactions in the etiology of conduct disorder and antisocial behavior. This study examined whether adolescent conduct disorder and adult antisocial behavior were related to the dopamine D2 receptor polymorphism (DRD2) and the dopamine D4 receptor polymorphism (DRD4).

**Methods:**

A sample of 872 male participants from the National Longitudinal Study of Adolescent Health (Add Health) completed self-report questionnaires that tapped adolescent conduct disorder and adult antisocial behavior. DNA was genotyped for DRD2 and DRD4.

**Results:**

Multivariate regression analysis revealed that neither DRD2 nor DRD4 had significant independent effects on conduct disorder or antisocial behavior. However, DRD2 interacted with DRD4 to predict variation in adolescent conduct disorder and in adult antisocial behavior.

**Conclusion:**

The results suggest that a gene × gene interaction between DRD2 and DRD4 is associated with the development of conduct disorder and adult antisocial behavior in males.

## Background

Adolescent conduct disorder (CD) and adult antisocial behavior are highly heritable phenotypes. Studies using twin designs and model-fitting techniques indicate that up to 85 percent of the variance in these disorders may be attributable to genetic influences[1,2]. However, the precise genetic polymorphisms implicated in the etiology of antisocial behaviors remain elusive. The most consistent findings suggest that genes related to the modulation of neurotransmitters may be associated with the development of maladaptive behaviors. For example, in a multivariate analysis of associations, Comings and his coauthors[3] examined 42 dopamine, serotonin, and norepinephrine genes on attention deficit hyperactivity disorder (ADHD), oppositional defiant disorder (ODD), and CD phenotypes. Their analyses revealed that CD was associated with multiple hormone and neuropeptide genes (CCK, CYP19, ESR1, and INS). In a similar vein, Caspi et al[4] examined whether the monoamine oxidase A (MAOA) polymorphism was associated with different types of antisocial conduct. Their analysis of 442 Caucasian males from the Dunedin Longitudinal Study revealed that MAOA did not have a significant direct effect on criminal or violent behaviors. However, MAOA interacted with childhood maltreatment to predict variation in criminal behavior. Other studies on MAOA have produced mixed results [5-7].

Taken together, empirical evidence suggests that antisocial conduct constitutes a polygenic phenotype, with the effects of some polymorphisms conditioned by the possession of other polymorphisms – that is, a gene × gene interaction[3]. However, we know of no research that has reported a gene × gene interaction on a CD behavioral phenotype. Limited evidence suggests that some genes and some proteins may act synergistically to create certain diseases and disorders[8]. MnSOD and GPX-1, for example, have been found to interact to increase the risk of developing breast cancer[9].

The most compelling evidence to suggest that gene × gene interactions may be implicated in the development of certain phenotypes comes from three studies. The first study, conducted by Noble and colleagues[10], examined the effect that two dopamine receptor genes (DRD2 and DRD4) had on personality traits. The results of their analysis revealed a significant interaction between minor variants of the DRD2 gene and the 7-repeat allele of DRD4 in the creation of novelty seeking. Another set of researchers attempting to replicate this finding failed to detect a significant gene × gene interaction[11].

The second study, carried out by Carrasco et al[12] examined the independent and interactive effects of DRD4 and DAT1 on ADHD in a sample of Chilean families. They employed a family-based discordant sib-pair analysis, which indicated that DRD4 and DAT1 failed to have significant and independent effects on ADHD. However, individuals who possessed both the 7-repeat allele of the DRD4 gene and the 10-repeat allele of the DAT1 gene were significantly more likely to be diagnosed with ADHD (odds-ratio = 12.71) when compared to those subjects with none or just one of these risk alleles.

In the third study, Eisenberg and his colleagues[13] analyzed data drawn from a sample of college students to estimate the effects of DRD2 and DRD4 on a behavioral measure of impulsivity. The results of their study revealed that DRD2 had a statistically significant main effect on impulsivity, but DRD4 did not. Additional analyses indicated that DRD2 and DRD4 interacted to predict variation in the measure of impulsivity.

Together these three studies provide some empirical evidence indicating that dopaminergic genes may interact to produce novelty-seeking, ADHD, and impulsivity. These findings are particularly important in selecting genetic polymorphisms that may also be implicated in the development of antisocial phenotypes because ADHD and impulsivity are highly comorbid with aggression and violence[14]. Moreover, several recent studies have revealed that the covariation among ADHD, impulsivity, conduct disorder, and oppositional defiant disorder is due, in large part, to shared genetic effects[15,16]. Thus genes that are associated with ADHD may also be the same genes that are associated with antisocial behavioral phenotypes.

Even though significant gene × gene interactions have not been extended to an antisocial behavioral phenotype, a number of lines of research converge to show that dopaminergic polymorphisms have independent effects on a wide array of maladaptive and antisocial phenotypes, such as compulsive gambling, alcohol consumption, and antisocial personality traits[3,17-19]. These studies suggest that dopaminergic genes may be etiologically related to conduct disorder and to antisocial behavior. From this research, two genetic polymorphisms have been identified as potentially important contributors to antisocial phenotypes: the dopamine D2 receptor gene (DRD2) and the dopamine D4 receptor gene (DRD4). DRD2 is a member of the D2 receptor family and has been mapped to chromosome 11 at location 11q23[20,21]. DRD2 codes for the D2 receptor and is found throughout the body, but especially in the striatum, the pituitary gland, the amygdala, the caudatus, the putamen, and other regions of the brain[22].

The A-1 allele of the DRD2 gene is considered the risk allele for antisocial phenotypes. Research investigating the functional role of the A-1 allele has found that carriers of this allele, in contrast to carriers of the A-2 allele, have fewer brain D2 dopamine receptors[23,24], have diminished glucose metabolism in the brain[25], are more attuned and responsive to stress[26], and exhibit reduced dopaminergic activity in the central nervous system[23]. As a result of the findings from these studies, the A-1 allele of DRD2 has been tagged as a contributor to the "reward deficiency syndrome" of the human body[27,28].

DRD4 has been mapped to chromosome 11 at location 11p15.5[29]. Similar to DRD2, DRD4 also belongs to the D2 dopamine family but manufactures the D4 dopamine receptor protein instead of the D2 dopamine receptor protein. The D4 dopamine receptor protein is found in areas of the brain that are responsible for the expression of emotions and for the stimulation of cognitive faculties[30]. Like other genes in the dopaminergic system, the DRD4 gene regulates attention processes, is partially responsible for motivation, and has been linked to exploratory behaviors[30]. The 7-repeat allele has been shown to mediate a blunted intracellular response to dopamine and may also encode a postsynaptic receptor that is subsensitive to dopamine[31]. The DRD4 polymorphism is one of the most promising candidate genes to many behavioral, psychiatric, and neuropsychological disorders[32].

In this study, we test whether DRD2 and DRD4 are associated with multiple measures of conduct disorder and with a measure of antisocial behavior. A considerable amount of research reveals, however, that complex phenotypes, such as antisocial behavior, are probably due to multiple genes acting not only independently, but also interactively. As a result, we also examine the possibility that DRD2 and DRD4 interact to increase the likelihood of evincing signs of antisocial behavior.

## Methods

### Study population

Subjects for this study come from the sibling-pairs sample of the National Longitudinal Study of Adolescent Health (Add Health). The Add Health is a prospective, nationally-representative sample of American adolescents in seventh through twelfth grade. Three rounds of interviews have been conducted thus far. The first wave of data was collected in 1994 when the participants were 11–19 years old. Over 90,000 respondents were administered a self-report survey at school. A random subsample of participants was then chosen to be interviewed at home and to be followed longitudinally. Nearly 20,700 adolescents and 17,700 of their primary caregivers (typically the mother) were interviewed at home. Approximately two years later, the second wave of questionnaires was completed by 14,738 participants. During 2001–2002, when the respondents were 18–27 years of age, the third wave of data was collected from 15,197 of the original Add Health participants. Detailed information about the Add Health research design and demographic statistics about the sample have been presented elsewhere[6,33].

The Add Health data also contain a subsample of sibling pairs. During wave 1 interviews, adolescents were asked whether they lived with a co-twin, a half-sibling, an unrelated sibling (e.g., a stepsibling), or a cousin. If they did live with a sibling, and if their sibling was between the ages of 11 and 20, then their sibling was asked to participate in the study. A probability sample of full siblings was also selected to be included in the sibling pairs sample[33]. Altogether, 5,470 siblings were included in the wave 1 sample, 4,984 were included in the wave 2 sample, and 4,356 were re-interviewed at wave 3[6].

During wave 3 interviews, a sample of N = 2,611 respondents was selected from the sibling pairs data and asked to submit samples of their DNA for genotyping. In line with prior research[6], we only used data that were available for males. We include both African American and non-Hispanic Caucasian males in our analytical data set (we include a dichotomous dummy variable controlling for race). Females were removed from the sample for two main reasons. First, for females there was very little variation in the dependent variables, with less than one percent of the sample of females scoring two standard deviations above the mean on the outcome measures. Second, preliminary analysis did not reveal any associations among DRD2, DRD4, and measures of antisocial behaviors for females. With this selection criteria, and after removing eighty-six cases that had missing data, and after removing one twin from each MZ twin pair, we were left with a final sample size of N = 872 male participants.

### Measures

The Add Health data contain a number of items that approximate some of the DSM-IV criteria for conduct disorder. We used these items to create a continuous conduct disorder scale for each of the three waves of data. At wave 1, respondents were asked how many times in the past 12 months had they gotten into a serious physical fight, had they hurt someone badly enough to need medical attention, had they used or threatened to use a weapon, and had they taken part in a group fight. At wave 2, adolescents were asked how many times in the past 12 months had they used or threatened to use a weapon, had they taken part in a group fight, and whether they had ever been initiated into a named gang (0 = no, 1 = yes). At wave 3, participants were asked how many times in the past 12 months had they threatened to use a weapon, had they taken part in a group fight, had they used a weapon in a fight, and had they ever been initiated into a named gang (0 = no, 1 = yes). Reponses to these items were coded as follows: 0 = never, 1 = 1 or 2 times, 2 = 3 or 4 times, 3 = 5 or more times. For each wave, answers to the items were summed together to form the conduct disorder scale at wave 1, the conduct disorder scale at wave 2, and the conduct disorder scale at wave 3[6].

Following prior research[6], we also created a lifetime conduct problems scale and a composite index of antisocial behavior. The lifetime conduct problems scale was created by summing the scores for the three conduct disorder scales and dividing by three. The resulting product indexed the average conduct disorder score across the three waves of data.

The composite index of antisocial behavior scale was developed by transforming the lifetime conduct problems measure into a standardized score (z-score). Respondents who scored more than 1.5 standard deviations units above the mean on this scale were assigned a value of "1," whereas participants scoring at or below 1.5 standard deviation units below the mean were assigned a value of "0." We also employed a one-item measure asking respondents to indicate whether they had ever been convicted of a felony. Participants who responded affirmatively were assigned a value of "1"; all other respondents were scored with a value of "0." Finally, the two items (i.e., the transformed conduct disorder scale and the violent conviction scale) were added together to form the composite index of antisocial behavior[6]. Scores on this scale ranged from zero to two. Table 1 provides descriptive information about each of the scales.

**Table 1:** Descriptive statistics for the antisocial phenotype measures

Measure	Mean	Standard Deviation	Range
Conduct disorder at wave 1	1.28	1.95	0–12
Conduct disorder at wave 2	.33	.82	0–7
Conduct disorder at wave 3	.40	.77	0–7
Lifetime conduct problems	.67	.91	0–5.67
Composite antisocial behavior index	.08	.29	0–2

Race (0 = Caucasian, non-Hispanic, 1 = African American) and age (measured in years) were included in the analysis as control variables.

### Genotyping

At wave 3, buccal cells were collected from respondents and genotyped for DRD2 and DRD4. In the 3' untranslated region of the DRD2 polymorphism is the site of TaqIA, which was genotyped as a single nucleotide polymorphism (SNP). Geneticists working at the Institute for Behavioral Genetics at the University of Colorado originated an SNP assay by employing the Applied Biosystem's "Taqman^© ^Assays by Design™ for SNP Genotyping Service"[34]. To genotype the DRD2 TaqIA polymorphism, the following primers and probes were used: forward primer, 5'-GTGCAGCTCACTCCATCCT-3', reverse primer, 5'-GCAACACAGCCATCCTCAAAG-3', probe 1, 5'VIC-CCTGCCT***T***GACCAGC-NFQMGB-3' and probe 2, 5'-FAM-CTGCCT***C***GACCAGC-NFQMCB-3'[34]. The DRD2 polymorphisms were scored by two independent observers, where the T-probe signal corresponded to the TaqIA-1 allele and the C-probe signal corresponded to the TaqIA-2 allele. The DRD2 alleles were then summed together to index the number of A-1 alleles that each subject possessed. Based on this nomenclature, 58 percent of the sample possessed zero A-1 alleles, 35 percent of the sample possessed one A-1 allele, and 7 percent of the sample possessed two A-1 alleles. The allelic distribution of the A-1 allele was examined by race, which revealed that African Americans possessed significantly more A-1 alleles than non-Hispanic Caucasians (χ^2 ^= 19.29, df = 2, *p *< .001).

The dopamine D4 receptor gene (DRD4) is a highly polymorphic gene that consists of a 48 base pair VNTR that can be repeated 2 to 11 times, although 2, 4, and 7 are the most common alleles. DRD4 was amplified by using the two proceeding primer sequences: forward, 5'-AGGACCCTCATGGCCTTG-3' (fluorescently labeled), and reverse, 5'-GCGACTACGTGGTCTACTCG-3'. This assay resulted in PCR products of 379, 427, 475, 523, 571, 619, 667, 715, 763, and 811 base pairs[19]. The two most common alleles in the Add Health sample were the 4-repeat and the 7-repeat. Following prior research[19], we pooled together the 379 (2R), 427 (3R), 475 (4R), 523 (5R), and 571 (6R) bp alleles and pooled together the 619 (7R), 667 (8R), 715 (9R), and 763 (10R) bp alleles. Subjects were then classified into three groups based on the number of 7R alleles that each subject possessed. Sixty-four percent of the sample possessed zero 7R alleles, 31 percent of the sample possessed one 7R allele, and 5 percent of the sample possessed two 7R alleles. Racial differences in the distribution of 7R alleles was examined and the results revealed that African Americans possessed significantly more 7R alleles than non-Hispanic Caucasians (χ^2 ^= 5.94, df = 2, *p *= .051).

### Statistical analysis

Since the dependent variables used in our analyses are count measures and therefore represent a Poisson distribution, we employed negative binomial regression analyses to examine the independent and interactive effects of DRD2 and DRD4 on conduct disorder and antisocial behavior. All models were calculated using Huber/White variance estimates to account for the non-independence in some of the observations. We estimated five different statistical models: one for each of the three conduct disorder scales, one for the lifetime conduct problems scale, and one for the composite index of antisocial behavior. To examine the possibility that the two genes may interact with each other, we created an interaction term by multiplying DRD2 and DRD4. In order to reduce problems of collinearity, the main effect terms were mean centered prior to constructing the gene × gene interaction term[35]. We also calculated variance inflation factors and tolerance limits to ensure that our analyses were not affected by multicollinearity. Based on these diagnostic estimates, no violations were detected. Hardy-Weinberg equilibrium was fulfilled for both DRD2 (χ^2 ^= 1.86, df = 1, *p *= .173) and DRD4 (χ^2 ^= 2.20, df = 1, *p *= .138) in the Add Health sample and we had adequate statistical power (80%) to detect an effect size of .35.

## Results

Table 2 contains the results of the negative binomial regression equations employing the three conduct disorder scales as dependent variables. The left hand side of the table reveals that DRD2 has a marginally significant effect on conduct disorder (*b *= .141, *p *= .059), whereas DRD4 does not exert an independent effect on the wave 1 conduct disorder scale (*b *= .036, *p *= .656). However, the interaction between DRD2 and DRD4 is a statistically significant predictor of conduct problems (*b *= .269, *p *= .030), even after partitioning out the effects of age and race.

**Table 2:** The independent and interactive effects of DRD2 and DRD4 on conduct disorder

	Conduct Disorder at Wave 1				Conduct Disorder at Wave 2				Conduct Disorder at Wave 3			
	*b*	SE	*z*	*p*	*b*	SE	*z*	*p*	*b*	SE	*z*	*p*
												
Polymorphisms												
DRD2	.141	.07	1.89	.059	.102	.12	.83	.408	.030	.10	.29	.769
DRD4	.036	.08	.45	.656	.014	.12	.11	.909	-.023	.10	-.24	.812
DRD2 × DRD4	.269	.12	2.17	.030	.486	.19	2.55	.011	.322	.15	2.07	.039
												
Control variables												
Age	-.055	.03	-1.80	.072	-.038	.05	-.83	.407	-.121	.04	-3.38	.001
Race	.246	.12	2.08	.038	.643	.18	3.56	.001	.317	.15	2.07	.039

Individuals possessing the A-1 allele *and *the 7R allele are significantly more likely to develop conduct problems in adolescence.

Similar results were garnered for the equations predicting the wave 2 conduct disorder scale. DRD2 (*b *= .102, *p *= .408) and DRD4 (*b *= .014, *p *= .909) fail to maintain a statistically significant association with conduct problems. In contrast, the interaction between these two genes has a statistically significant effect (*b *= .486, *p *= .011) on conduct disorder. The pattern of finding for the wave 3 conduct disorder scale mirrors those for the wave 2 conduct disorder scale; DRD2 (*b *= .030, *p *= .769) and DRD4 (*b *= -.023, *p *= .812) fail to have a significant effect on conduct disorder, but the interaction between DRD2 and DRD4 has a significant effect (*b *= .322, *p *= .039) on the wave 3 conduct disorder scale.

Table 3 presents the results of the negative binomial regression equations predicting the lifetime conduct problems scale and the composite index of antisocial behavior. The coefficients displayed under the first column show that DRD2 (*b *= .096, *p *= .127) and DRD4 (*b *= .041, *p *= .575) do not have significant independent effects on lifetime conduct problems scale. However, the interaction between DRD2 and DRD4 has a significant effect (*b *= .296, *p *= .003) on the lifetime conduct problems scale.

**Table 3:** The independent and interactive effects of DRD2 and DRD4 on lifetime conduct problems and composite index of antisocial behavior

	Lifetime Conduct Problems				Composite Index of Antisocial Behavior			
	*b*	SE	*z*	*p*	*b*	SE	*z*	*p*
								
Polymorphisms								
DRD2	.010	.06	1.52	.127	.205	.16	1.31	.189
DRD4	.041	.07	.56	.575	-.113	.22	-.52	.600
DRD2 × DRD4	.296	.10	3.00	.003	.656	.23	2.85	.004
								
Control variables								
Age	-.084	.03	-3.18	.001	-.080	.06	-1.25	.210
Race	.338	.11	3.14	.002	.557	.26	2.11	.035

The second column in Table 3 uses the composite index of antisocial behavior as the dependent variable. DRD2 (*b *= .205, *p *= .189) and DRD4 (*b *= -.113, *p *= .600) do not maintain significant associations with the measure of antisocial behavior. However, the interaction between DRD2 and DRD4 again emerges as a significant predictor (*b *= .656, *p *= .004).

## Discussion and conclusion

We examined whether two dopaminergic polymorphisms – DRD2 and DRD4 – were related to conduct disorder and adult antisocial behavior. The results of the statistical models revealed that DRD2 and DRD4 did not have consistent main effects on antisocial phenotypes. However, for all five of the equations estimated, DRD2 interacted with DRD4 to predict variation in the conduct disorder scales and variation in the composite index of antisocial behavior. These findings thus provide initial evidence of a gene × gene interaction on conduct disorder and antisocial behavior in a sample of males. The results are consistent with other biological studies which point to the likely interaction of several genes in producing complex disorders such as autism[36] and schizophrenia[37].

The statistical analyses employed in this study were only able to demonstrate that a correlation existed between the antisocial phenotypes and the gene × gene interaction. Correlations, of course, do not imply causation and standard statistical techniques, such as the ones used here, are unable to provide much information about the causal processes that might lead from the gene × gene interaction to conduct disorder or to adult antisocial behavior. Recent theoretical work by Sagvolden and his colleagues[38], however, is useful in shedding some light on how dopaminergic polymorphisms, such as DRD2 and DRD4, may lead to various forms of psychopathology. According to the dynamic developmental behavioral theory proposed by Sagvolden et al, a hypofunctioning mesolimbic dopamine system is largely responsible for producing alterations in behavioral reinforcement and extinction, which, in turn, disrupts learning processes. These disrupted learning processes coupled to a hypofunctioning mesocortical dopamine pathway can cause the emergence of ADHD and ADHD-like symptoms.

Although Sagvolden et al's theory was designed to provide a causal explanation to the etiology of ADHD, it is possible that this theoretical perspective may extend to non-ADHD phenotypes, such as antisocial behavior. Sagvolden et al, for example, often make reference to the fact that altered dopaminergic functioning has effects on behavioral disinhibition. There is now an impressive amount of research revealing that many types of antisocial behaviors, including violence and aggression, are linked to an inability to control impulses and to a lack of behavioral inhibition [39]. Perhaps Sagvolden et al's model is much broader in scope, where ADHD is just one symptom of a larger constellation of phenotypes – including conduct disorder and adult antisocial behavior – that can be explained by the dynamic developmental behavioral theory.

With this in mind, it is important to touch upon the main limitations of our study. First, the Add Health data did not contain information about a clinical diagnosis of conduct disorder, thus we used measures that approximated conduct disorder. The measures used to construct the conduct disorder scales were based largely on overt forms of physical violence. Of course, conduct disorder also includes more covert types of behavior, such as stealing and vandalism. Although prior researchers examining genetic influences with the Add Health data have used a similar strategy and similar items to tap conduct disorder[6], it is possible that the gene × gene interaction is only associated with overt physical aggression. Extant research has revealed that heritability estimates fluctuate depending on whether aggression is measured through covert behaviors or overt behaviors[40]. As a result, replication studies should address this possibility and examine whether the gene × gene interaction is found for covert antisocial phenotypes.

On a related note, conduct disorder is often comorbid with drug use and sensation seeking. At the same time, DRD2 and DRD4 have been found to be related these different phenotypes[11,41-43], thus raising the possibility that the gene × gene interaction is spurious, with the confounding factors being drug dependence. Research is needed to examine whether the interaction between DRD2 and DRD4 is observed in other samples and, if so, if other phenotypes (e.g., drug use) can explain this association.

It is also important to point out that only a subsample of respondents from the Add Health data submitted sample of their DNA for genotyping. This raises the possibility that the sample used in the analyses is not a nationally-representative cross-section of American males. Similarly, thus far the Add Health respondents have only been tracked through young adulthood. Whether the gene × gene interaction would be detected later in life is an empirical question that subsequent research needs to investigate.

We close by noting that although our findings point to the possibility that antisocial phenotypes may be partially the result of an interaction between DRD2 and DRD4, it is possible that other genes that are in close linkage with DRD2 and DRD4 may actually be driving this effect. Analyses that examine haplotype blocks would begin to elucidate the true causal genetic variants to behavioral phenotypes. Future research will have to explore these possibilities. As for now, our research draws attention to the importance of analyzing whether multiple polymorphisms work in concert to bring about phenotypic differences.

## Competing interests

The author(s) declare that they have no competing interests.

## Authors' contributions

KMB analyzed the data, interpreted the findings, and wrote and revised the manuscript. JPW helped interpret the findings and write the manuscript. MD assisted in writing and revising the manuscript. AW helped write the manuscript. MGV helped with data analysis and with revising the manuscript. DB assisted in revising the paper. JV assisted in revising the paper. All authors read and approved the final draft of the manuscript.
